# Targeting astrocytes polarization after spinal cord injury: a promising direction

**DOI:** 10.3389/fncel.2024.1478741

**Published:** 2024-10-16

**Authors:** Helin Li, Ying Liu, Yucao Sun, Hangyu Guo, Shiyan Lv, Wenhui Guo, Jiyu Ren, Yufu Wang, Jianing Zu, Jinglong Yan, Nanxiang Wang

**Affiliations:** ^1^Department of Orthopedic Surgery, The Second Affiliated Hospital, Harbin Medical University, Harbin, China; ^2^Department of Ultrasound, Harbin Medical University Cancer Hospital, Harbin, China

**Keywords:** SCI, astrocyte, A1 astrocytes, A2 astrocytes, polarization

## Abstract

Spinal cord injury (SCI) is a serious neurological injury that causes severe trauma to motor and sensory functions. Although long considered incurable, recent research has brought new hope for functional recovery from SCI. After SCI, astrocytes are activated into many polarization states. Here we discuss the two most important classical phenotypes: the ‘A1’ neurotoxic phenotype and the ‘A2’ neuroprotective phenotype, with A1 astrocytes being neurotoxic and impeding neurorecovery, and A2 astrocytes being neuroprotective. This paper discusses the changes in astrocyte responsiveness after SCI and the pros and cons of their polarization in SCI. It also elucidates the feasibility of astrocyte polarization as a therapeutic target for neuroprotection. In the future, multiple intervention strategies targeting astrocyte polarization are expected to gain wider clinical application, ultimately improving motor-sensory function and quality of life in SCI patients.

## 1 Introduction

Spinal Cord Injury (SCI) is a severe neurological injury that results in a profound neurological impairment that culminates in enduring or irreversible deficits in motor, sensory, and autonomic functionalities (Ahuja et al., 2017; Maier and Schwab, 2006; Anjum et al., 2020; Sterner and Sterner, 2022). The worldwide prevalence of SCI is notably high, averaging between 30.0 to 40.0 incidents per million in the United States and 23.7 to 60.6 incidents per million in China, as indicated by recent studies (Singh et al., 2014; Jazayeri et al., 2015). SCI not only has a severe impact on patient’s quality of life but also poses unprecedented challenges in healthcare and rehabilitation. Despite significant advances in neuroprotection, regeneration, and repair in recent years, medical science and technology, functional recovery following SCI still faces many obstacles and difficulties (Ahuja et al., 2017).

Astrocytes, the most abundant type of glial cell in the central nervous system (CNS) (Lawrence et al., 2023), have a variety of critical biological functions, including supporting trophic neurons, regulating synaptic activity and neuronal electrical activity, and maintaining the integrity of the blood–brain barrier and blood-spinal cord barrier (Seifert et al., 2006; Halassa et al., 2007). In the resting state, astrocytes exhibit a “homeostatic” phenotype, but in SCI or other pathological states, astrocytes become activated and polarized, and their phenotypic and functional changes are critical for repair and regeneration following SCI. This review is intended to explore the impact of polarized astrocytes on the repair of spinal cord injury and to elucidate the feasibility of utilizing astrocyte polarization as a therapeutic target for neuroprotection.

## 2 Astrocyte response after SCI

The response of astrocytes following SCI is extremely complex and multifaceted, involving significant morphological and functional changes. The extent of their response is influenced by various cell surface DAMPS receptors and pro-inflammatory cytokines and chemokines (Sofroniew, 2014b). After the injury, astrocytes are rapidly activated and undergo a series of biological variations, including significant hypertrophy of the cyton and protrusions, proliferation, and migration (Sofroniew, 2014a; Anderson et al., 2014; Sofroniew, 2009; Koyama, 2014). At the same time, the function of these activated astrocytes undergoes drastic variations, and these functional variations are crucial for repairing damage and restoring function. Relevant studies have identified multiple polarization possibilities for astrocytes. However, we found that the A1/A2 phenotype may be more useful in describing the state and function of reactive astrocytes in SCI, as well as being supported by a large body of research, so this paper focuses on the A1/A2 phenotype. In response to ischemia, astrocytes undergo polarization and assume a neuroprotective/A2 phenotype (Liddelow and Barres, 2017; Zamanian et al., 2012; Su et al., 2019). Characteristic hallmarks include S100a10, Clcf1, Ptx3, Emp1, and S1pr3 (Zamanian et al., 2012; Liddelow and Barres, 2017). These type A2 astrocytes exert neuroprotective functions via the generation of anti-inflammatory cytokines and neurotrophic factors that promote tissue repair and regeneration (Liddelow et al., 2017; Jang et al., 2013; Zamanian et al., 2012; Wang et al., 2021b). In contrast, specific cytokines secreted by microglia exposed to lipopolysaccharide (LPS), neuroinflammation, etc. induce a cytotoxic A1 astrocyte (Liddelow and Barres, 2017; Liddelow et al., 2017). Characteristic markers are complement C3, iNOS, SerpinG1, and H2d1 (Zamanian et al., 2012; Kisucká et al., 2021). Out of these, C3 is the most critical biomarker for A1 astrocytes, which are engaged in a variety of crucial processes. These type A1 astrocytes are incapable of promoting neuronal survival and growth, synapse formation, and phagocytosis (Wang et al., 2023), as well as strongly upregulate a variety of genes that are detrimental to synapses (e.g., complement system genes) (Liddelow and Barres, 2017), even triggering neuronal death (Chang et al., 2023).

## 3 Pros and cons of astrocyte polarization after SCI

Understanding these two phenotypes’ biological functions can help delve into repair strategies for SCI. Due to the distinctly different functions of A1/A2 astrocytes, their roles in functional recovery from SCI are also opposing:

### 3.1 A2 astrocytes promote neuronal survival and functional recovery through multiple mechanisms

#### 3.1.1 Secretion of neurotrophic factors

**Figure 1:**
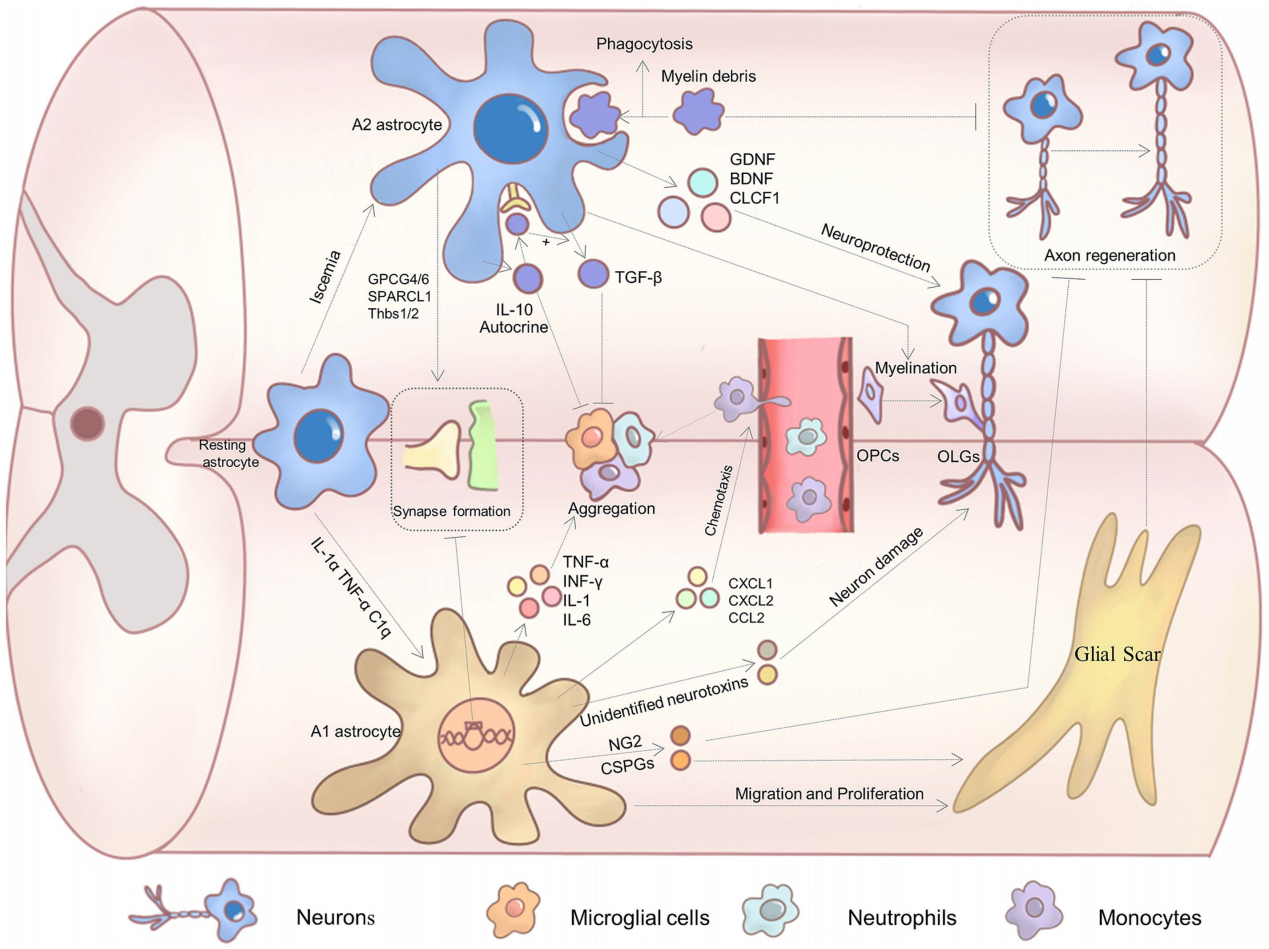
The effect of astrocyte polarization on recovery from SCI.

Astrocytes of this phenotype secrete a good deal of neurotrophic factors, such as BDNF, GDNF, CLCF1, and HIF (Zamanian et al., 2012; Liu L. R. et al., 2020). They confer great neuroprotective functions to A2 astrocytes. For example, BDNF promotes neuronal survival, synaptic plasticity, and axon growth by binding to its receptor TrKB and activating the downstream MAPK/ERK signaling pathway (Wang et al., 2024; Liang et al., 2019). Meanwhile, GDNF acts mainly through the Ret receptor to support motor and sensory neuron survival (Durbec et al., 1996). *In vitro* co-culture of A2 astrocytes with neurons significantly inhibited high glutamate-induced neuronal apoptosis, significantly reduced pro-apoptotic proteins such as caspase 3, caspase 9, and Bax, and promoted neuronal dendritic arborization (Chang et al., 2023). The impact of A2 astrocytes on neurons is likely to be mediated by the secretion of certain neurotrophic factors. These trophic factors play a pivotal role in SCI (Figure 1).

#### 3.1.2 Regulation of inflammatory response

Excessive inflammatory response following SCI can cause severe tissue damage (Xu L. et al., 2018). Astrocytes of the neuroprotective phenotype can secrete anti-inflammatory factors (IL-10 and TGF-*β*), which regulate and inhibit excessive local inflammatory responses (Zhang et al., 2020b). For example, IL-10 can limit immunoreaction, promote myelin regeneration, and facilitate neuronal repair (Yang et al., 2009; Hellenbrand et al., 2019). IL-10 also causes A2 astrocytes to secrete TGF-β through autocrine means and reduces microglia activation (Norden et al., 2014). It has been demonstrated that A2 astrocytes are also capable of inhibiting the accumulation of reactive microglials (Chang et al., 2023). These findings offer new insights into the mechanisms by which astrocytes regulate microglials in the context of inflammatory conditions. Meanwhile, this interaction suggests that polarized astrocytes after SCI may be involved in regulating multiple cellular activities.

#### 3.1.3 Removal of harmful substances

A2 astrocytes play an instrumental role in the alleviation of a toxic environment in the injured area through the phagocytosis and clearance of harmful agents, such as myelin fragments and metabolites (Morizawa et al., 2017). Furthermore, myelin fragments have been observed to contain axon growth inhibitors, which can impede axon regeneration (Schwab, 2010). The removal of such inhibitory substances can facilitate more conducive conditions for neuronal repair and regeneration.

#### 3.1.4 Involvement in the processes of synapse formation and repair and promotion of myelin regeneration

Astrocytes with neuroprotective properties have been demonstrated to secrete GPCG4/6 (Allen et al., 2012), SPARCL1 (Kucukdereli et al., 2011) and pro-synaptic platelet-responsive proteins (e.g., Thbs1/2), which promote synapse formation (Christopherson et al., 2005) and accelerate synapse repair following SCI (Chan et al., 2019; Eroglu et al., 2009). Myelin regeneration is a vital component of the recovery process following a central nervous system injury (Psachoulia et al., 2016; Lee et al., 2015). The transplantation of A2 astrocytes has been shown to enhance myelin regeneration at the lesion site in SCI models, indicating that A2 astrocytes may facilitate the formation of mature myelinating oligodendrocytes (Chang et al., 2023).

A2 astrocytes are implicated in the pathophysiology of SCI through multiple pathways, ultimately exerting a supportive influence on neurons and promoting regeneration.

### 3.2 A1 astrocytes impede functional recovery through the following mechanisms

#### 3.2.1 Secretion of pro-inflammatory factors and chemokines

A1 astrocytes secrete significant quantities of inflammatory factors, including TNF-*α*, IL-1β, IFN-*γ*, IL-1, and IL-6 (Li et al., 2019; Jang et al., 2013), which have been demonstrated to markedly promote the activation of inflammatory cells, resulting in extensive inflammatory cell infiltration. A1 astrocytes also rapidly synthesize many inflammatory chemokines, such as CCL2, CXCL1, and CXCL2. These chemokines facilitate the recruitment of blood-derived immune cells (e.g., type I “inflammatory” monocytes and neutrophils) to infiltrate the lesion site, thereby exacerbating inflammatory cell aggregation and activation, thus creating an even harsher microenvironment (Pineau et al., 2010). At the same time, it has been demonstrated that IL-1R is a key regulator of the expression of these chemokines by astrocytes after SCI, regulating the secretion of chemokines from A1 astrocytes (Pineau et al., 2010). The intense inflammatory response caused by the above factors exacerbates damage in the SCI region.

#### 3.2.2 Formation of glial scars

Following the SCI, A2 astrocytes proliferate and secrete plenty of extracellular matrix components. A significant consequence of SCI is the proliferation and migration of a considerable number of reactive astrocytes, which subsequently form a multitude of glial scars at the margins of the lesion area. While moderate glial scars can form a protective barrier against further expansion of the lesion area, these glial scars form a substantial physical barrier that impedes axon growth (Cregg et al., 2014; O'Shea et al., 2017). Additionally, astrocytes secrete axon inhibitory factors, such as CSPGs and NG2, further inhibiting axon elongation (Silver and Miller, 2004). CSPGs and NG2 proteoglycans can induce neurite retraction and growth cone collapse, inhibiting axon growth and regeneration (McKeon et al., 1991; Ughrin et al., 2003). Related studies have demonstrated that the utilization of specific enzymes to induce CSPG degradation or impede its formation can markedly enhance axon growth and regeneration (Faissner et al., 1994; Smith-Thomas et al., 1995; Tom and Houlé, 2008). What is noteworthy is that STAT3 has been demonstrated to play a pivotal role in astrocyte glial scar formation, and pSTAT3 was markedly elevated following SCI. In STAT3-CKO mice, there was a striking reduction in GFAP up-regulation in the injured region, and astrocytes were unable to undergo hypertrophy, resulting in a considerable diminution in astrocyte scar formation (Herrmann et al., 2008). These findings imply that STAT3 may be a critical regulator of reactive astrogliosis. Inhibition of the aforementioned factors may offer a novel avenue for promoting axonal regeneration following SCI. Conversely, Anderson et al. (2016) demonstrated that astrocyte glial scarring facilitates rather than impedes CNS axonal regeneration. This is contrary to the prevailing dogma. However, Silver pointed out the limitations of Anderson’s view because glial scarring is made up of multiple components, not just astrocytes (Silver, 2016). Consequently, the role of glial scarring in axonal regeneration remains a topic of contention, and further substantiation is necessary.

#### 3.2.3 Neurotoxic impact

A subset of astrocytes undergo a polarization shift toward the A1 phenotype in the aftermath of the SCI. This transition results in the loss of their capacity to promote neuronal survival and growth and instead renders them neurotoxic. They are capable of rapidly killing mature neurons and differentiated oligodendrocytes (Liu et al., 2022). The co-culturing of reactive A1 astrocytes with retinal ganglion cells (RGCs) demonstrated that RGCs undergo rapid death in higher concentrations of A1 medium/ACM (Liddelow et al., 2017). Similarly, the co-culturing of A1 astrocytes with spinal motor neurons exhibited a mere 20% survival rate of the latter (Liddelow et al., 2017). These findings imply that A1 astrocytes might secrete some kind of soluble neurotoxin that induces neuronal death. Liang et al. (2023) demonstrated that neurotoxic A1 astrocytes induce neuronal ferroptosis-associated lipid peroxidation through the secretion of CXCL10 and CXCR3. Bi et al. (2013) and Jung et al. (2023) observed that A1 astrocytes induced neuronal damage by secreting the neurotoxic LCN2 (Lipocalin-2). Furthermore, LCN2 has been proven to enhance the formation of canonical NLRP3 and the production of damaging substances such as IL-1β following SCI (Müller et al., 2023). Therefore, the hypothesis that A1 astrocytes produce a neurotoxin is further substantiated, and the specific neurotoxin or some neurotoxins that play a pivotal role in this process require further investigation. Furthermore, A1 astrocytes have been demonstrated to impede the proliferation of neural stem cells. When primary NSCs were exposed to neurotoxic astrocyte culture supernatant (astrocyte-conditioned medium, ACM), cell viability assays demonstrated that ACM significantly impeded the proliferation of NSCs (Qian et al., 2024). Furthermore, A1 astrocytes were found to upregulate the expression of numerous classical neurotoxin genes, leading to synaptic disruption (Miyamoto et al., 2020). In conclusion, A1 astrocytes are potent neurotoxins and have deleterious effects on SCI recovery.

Following the SCI, astrocytes undergo a phenotypic transformation, and their function is dual. Astrocyte polarization is likewise a complex process involving multiple genes and factors. Nevertheless, numerous studies have demonstrated that NF-κB and STAT3 are involved in a multitude of polarization pathways (Ageeva et al., 2024; Xu X. et al., 2018; Wang and Li, 2023; Li et al., 2023; Wang et al., 2018; Li et al., 2021; Zhang et al., 2021; Su et al., 2019). It is extremely likely that they are pivotal molecules in astrocyte polarization, regulating astrocyte phenotypic transformation. Furthermore, more recent and more detailed studies have demonstrated that soluble adenylyl cyclase and regional cyclic adenosine monophosphate in reactive astrocytes can serve as molecular switches for neuroprotective astrocyte reactivity, and potentially be a target for inhibiting microglial and A1 astrocyte activation (Cameron et al., 2024). The study also revealed that A2 astrocytes likely act upstream of the pathway that A1 astrocytes are activated by IL-1α, TNFα, and C1q, inhibiting the recruitment of detrimental microglia and preventing C3-positive A1 astrocytes from activating and neuronal death. This study is the first to propose an effect between A1 and A2 astrocytes and extends the complex neuroglia-neuroglia regulation. The identified key molecules provide a robust molecular basis for targeting astrocyte polarization treatments after SCI in the future.

## 4 Targeted astrocyte polarization after SCI

Following the SCI, astrocytes undergo a phenotypic transformation. Type A2 astrocytes facilitate neurological recovery by supporting neuronal survival, promoting axonal regeneration, and modulating inflammatory responses. In contrast, type A1 astrocytes impede spinal cord recovery by exacerbating inflammatory responses, forming glial scars, and exerting toxic effects. An in-depth study of the function of these two phenotypes gives us a clear direction to develop an effective therapeutic strategy: inhibiting A1 astrocytes or promoting the polarization of A2 astrocytes.

### 4.1 Pharmacological intervention

A substantial body of evidence from basic experiments indicates that the administration of particular drugs can influence the phenotypic transformation of astrocytes, which is conducive to the recuperation process following SCI. For example, the classical drug methylprednisolone has been demonstrated to exert a protective effect on neurons following traumatic SCI by inhibiting A1 astrocyte activation (Zou et al., 2021). As a potential protective agent induced by stress conditions in the organism, Sestrin2 has been demonstrated to inhibit A1 astrocyte activation. Further exploration revealed that Sestrin2 significantly up-regulated autophagy markers, such as Beclin1 and LC3-II, and mitochondrial autophagy biomarkers, PINK1 and Parkin. Furthermore, the autophagy inducer, rapamycin, inhibits GFAP and iNOS proteins and C3 mRNA levels. This indicates that autophagy or mitochondrial autophagy may be implicated in A1 astrocyte transformation and astrocyte inflammation, establishing a correlation between Sestrin2 and autophagy. Specifically, the inhibition of A1 astrocyte transformation by Sestrin2 is achieved by increasing autophagy levels (Pan et al., 2024). It is noteworthy that autophagy also appears to be involved in regulating the production of CSPGs. Studies have demonstrated that autophagy inhibits the production of CSPGs, thereby facilitating axonal regeneration (Alizadeh et al., 2021). Targeting autophagy might play a beneficial role in SCI recovery through multiple pathways. Meanwhile, intravascular injection of recombinant prokineticin 2/rPK2 selectively promoted astrocyte polarization to an A2 phenotype and induced STAT3 phosphorylation (Ma et al., 2020). Blockade of the Notch pathway with *γ*-secretase blockers (DAPT) alleviates A1 astrocyte-induced neuronal apoptosis and axonal damage (Qian et al., 2019). Interestingly, telmisartan, which has been used for many years in the treatment of hypertension, is also involved in the polarization process. Studies have confirmed that telmisartan inhibits microglia-induced A1 astrocyte activation and restricts the extent of the inflammatory response by degrading p65 (Quan et al., 2023). Furthermore, peroxisome proliferator-activated receptor gamma (PPARγ) antagonists have been demonstrated to reverse the neuroprotective effects of telmisartan; telmisartan may act through PPARγ. As classical neuroprotectants, gangliosides are of considerable preclinical and clinical value in various central nervous systems. Recent studies have shown that when co-cultures of astrocytes and neurons are treated with GM1, the neurons upregulate the expression of a variety of neuroprotective genes. It is speculated that GM1 may promote the polarization of astrocytes toward the A2 phenotype and thus neuroprotection (Finsterwald et al., 2021). Glucagon-like peptide-1 receptor (GLP1R) agonists have been demonstrated to be potential neuroprotective agents in a variety of CNS disorders (Harkavyi and Whitton, 2010; Athauda and Foltynie, 2016) and recent evidence indicates that NLY01 (a GLP1R agonist) directly prevents the microglia-mediated conversion of astrocytes to the A1 phenotype and is neuroprotective (Yun et al., 2018). Other drugs are attempting to modulate astrocyte polarization. However, it is important to recognize that when targeting a molecule for drug therapy, the molecule may be expressed in a wide range of cells and the impact is likely to be multiplicative, so it is essential to target astrocytes and develop drugs in a targeted manner.

### 4.2 Cell therapy

Cell transplantation has emerged as a viable method to promote repair following SCI (Assinck et al., 2017). Some studies are in phase I clinical trials (Curtis et al., 2018). By transplanting neuroprotective astrocytes or functional exosomes, a microenvironment favorable for nerve regeneration can be created at the injury site, thus promoting functional recovery after SCI (Liu et al., 2019). Recently, it has been demonstrated that when A2 astrocytes were selectively transplanted into spinal cord lesions after SCI, there was a reduction in the accumulation of glial scar, and more neurofilaments and myelin structures were detected (Chang et al., 2023). This direct transplantation experiment fully demonstrated the promise of cell therapy. Zhang J. et al. (2024) found that administration of M2 microglia-derived exosomes/M2-EXOs suppressed A1 astrocyte activation and promoted neuronal survival and axonal preservation in SCI mice. In contrast, extracellular vesicles/EVs derived from hypoxia-pretreated BMSCs regulated the astrocyte phenotype through the miR-21/JAK2/STAT3 pathway, promoted the conversion of A1 astrocytes to A2 astrocytes and facilitated recovery from SCI (Yang et al., 2024). Neuron-derived exosomes promoted functional recovery and reduced lesion volume after SCI and suppressed the activation of neurotoxic astrocytes. miRNA array analysis further revealed that miR-124-3p was most highly enriched in neuron-derived exosomes, leading to the conjecture that miR-124-3p dominates the activation of A1 astrocytes (Jiang et al., 2020). The low immunogenicity and robust biocompatibility and stability of exosomes determine their important therapeutic potential. Cell therapy has emerged as a promising therapeutic modality.

### 4.3 Biomaterials

Materials such as nanomaterials and hydrogels have been a hot research topic in recent years for the treatment of various diseases, and repair of the CNS, providing a new platform for stem cell and growth factor therapy. The important role of drug-loaded nanomaterials in modulating microglial activation has been well demonstrated in previous studies. For example, minocycline entrapped in NPs consisting of polymers based on poly *ε*-caprolactone and polyethylene glycol was demonstrated to attenuate the activation and proliferation of microglia around the lesion site of SCI, with a sustained reduction in the expression of the pro-inflammatory cytokine IL-6 and the expression of CD68 at the lesion site (Papa et al., 2013). Recent studies have also demonstrated the potential of polymeric nanomaterials for astrocyte polarization. The study by Vismara et al. (2020) observed that the selective internalization of nanostructured gels loaded with Rolipram (an NF-κB inhibitor) into astrocytes in mice significantly reduced the expression of the pro-inflammatory factors iNOS and Lcn2, and reversed the toxic effects of type A1 astrocytes on motor neurons *in vitro*. Polymeric nanoparticles have a high affinity for water, greater colloidal stability, and longer drug loading times to maximize therapeutic efficacy and minimize side effects. Chan et al. (2019) found that EGF-loaded hydrogels improved the survival of oxygen–glucose deprivation (OGD)-injured primary neurons by 3-fold compared to EGF alone and downregulated the expression of deleterious A1-like genes (Fbln5 and Rt1-S3) and upregulated advantageous A2-like genes (Clcf1, Tgm1, and Ptgs2) and upregulated the expression of PSD-95, the synaptophysin that promotes synapse formation, which promotes synaptic plasticity and exhibits clear neuroprotective properties. This growth factor-loaded hydrogel has excellent biocompatibility, degradability, and low immunogenicity. While providing a structural scaffold for tissues, the hydrogel can also be utilized as a slow-release carrier to continuously release protective drugs and accelerate the repair of damaged tissues, which is a great advantage in drug delivery. In addition, many previous studies have demonstrated the feasibility of drug-loaded hydrogels for neural stem cells and neuron differentiation, which promotes functional recovery following SCI (Liu et al., 2024; Song et al., 2024). In conclusion, biomaterials may be a significant direction for the future treatment of SCI.

### 4.4 Gene therapy

Gene therapy has been one of the hotspots of research in recent years. Specific gene editing technology (e.g., CRISPR/Cas9) is employed to regulate the expression of essential genes in astrocytes, thereby controlling their phenotypic transformation. Knockdown of Sirt1 with the CRISPR/Cas system resulted in the transformation of reactive astrocytes to the A2 phenotype (Zhang et al., 2022). Interestingly, Knockdown of microglial voltage-gated proton channel/Hv1 also reduced A1 astrocytes and increased A2 astrocytes, promoting synaptic and axonal remodeling. This indicates that we should take a more comprehensive view of astrocyte polarization, which may involve more dimensions and have multiple pathways for its transformation (Li et al., 2023). Herrmann et al. (2008) found that knockdown of the STAT3 gene in mice in the spinal cord injury model markedly inhibited the transformation of astrocytes to the A2 phenotype, resulting in a dramatic inflammatory response at the lesion site and an extension of the lesion volume. Therefore, appropriate promotion of STAT3 gene expression may be beneficial in restricting local inflammation. Noteworthy, we found that STAT3 plays an extremely significant role in the formation of glial scar, polarization, and axon formation of astrocytes, and acts as a pivotal triggering factor to control astrocytes, which was also confirmed in the study of STAT3 ablation after SCI (Okada et al., 2006), and therefore we believe that STAT3 is likely to become a critical therapeutic targeting molecule. Whereas exposure of mice to Mn at a young age significantly increased the amount of neurotoxic A1 astrocytes expressing C3 in the brain, in contrast to wild-type mice, knockout of I kappa B kinase 2/IKK2 (an upstream activator of NF-κB) significantly diminished A1 astrocytes in the brain (Hammond et al., 2020). Although there is a possibility of heterogeneity, all this evidence confirms the potential of gene therapy. With the application of AAV-NeuroD1 in patients, gene therapy has officially entered the clinical research phase. However, there are still ethical issues and the possibility of side effects, so gene therapy remains to be treated with caution. In addition, the phenotypic transformation of astrocytes can also be modulated by regulating the expression of non-coding RNAs. For example, silencing of miR-21 promotes the transformation of astrocytes from a neuroinvasive to a neuroprotective phenotype (Su et al., 2019; Liu et al., 2018) whereas miR-124 mediates Smad2 to suppress A1 astrocyte activation and facilitate recovery of spinal cord function (Su et al., 2019; Jiang et al., 2020). Interestingly, competing endogenous RNAs (ceRNAs) are also involved in the regulation of polarization. M2a macrophage conditioned medium (CM) significantly inhibited A1 astrocyte activation, whereas knockdown of ceRNA NEAT1 reversed this effect and significantly reduced protein levels of M2a biological markers, such as Arg-1 and YM-1, and anti-inflammatory cytokines, such as IL-4 and IL-13 levels (Liu et al., 2021), indicating that NEAT1 impedes A1 astrocyte polarization. The involvement of ceRNAs expands the direction of targeting astrocyte polarization, and in the future, we may be able to silence or upregulate certain key RNAs to truly promote the recovery of spinal cord function in patients in the clinic. In conclusion, we believe that gene therapy has an extremely promising future in the regulation of astrocyte polarization.

### 4.5 Photobiological therapy

Photobiology therapy has also been implicated in the regulation of astrocyte polarization. As a classical non-invasive physical therapy, photobiomodulation has numerous applications in various fields of medicine, with promising anti-inflammatory and tissue repair effects. Previous studies have found that photobiomodulation inhibits the high-level expression of pro-inflammatory factors and upregulates the expression of neurotrophic factors, as well as inhibiting microglial polarization toward neurotoxicity and promoting functional repair of SCI (Ju et al., 2023a; Sun et al., 2020; Zhang et al., 2020a). Recent findings have demonstrated that photobiomodulation can not only mediate Sox9 to downregulate CSPGs expression after SCI, but also inhibit A1 astrocyte activation and neurotoxicity to dorsal root ganglion (DRG) neurons, and upregulates bFGF and TGF-*β* expression, both of which regulate A1/A2 astrocyte transformation in a dose-dependent manner (Zhang Z. et al., 2024; Wang et al., 2021a; Wang et al., 2021b). The time course of A1/A2 astrocyte activation after SCI was determined by RNA sequencing and it was determined that astrocytes begin to polarize to the A1 phenotype at 7 days after SCI, whereas A2 polarization occurs earlier, although the degree of polarization appears to be lesser (Wang et al., 2021b). Analysis of the levels of polarization-specific transcripts will allow us to gain a deeper appreciation of the dynamic process of astrocyte polarization, providing a time basis for targeting polarization in the treatment of spinal cord injury. Noteworthy, STAT3 may be an extremely potential targeting molecule for the photobiological treatment of SCI, regardless of microglia or astrocyte (Ju et al., 2023b; Wang et al., 2021a). This emphasizes the role of STAT3 in astrocyte phenotypic transformation. Based on the above studies, photobiological therapy also provides a pathway to functional recovery in SCI.

### 4.6 Traditional Chinese medicine therapy

Certain herbal components have also been reported to modulate astrocyte polarization. Rao et al. (2024) observed that the combination of tetramethylpyrazine/TMPZ and ASG -IV can mediate the Sirt1/NF-κB axis to prevent A1 astrocyte activating, which promotes recovery from SCI. In the middle cerebral artery occlusion (MCAO) model, administration of Buyang Huanwu decoction reduced the activation of microglia and A1 astrocytes, greatly diminished the level of inflammatory factors, and promoted the expression of BDNF, which effectively alleviated ischemic stroke injury (Li et al., 2024). Honokiol were able to modulate the SIRT3-STAT3 axis to inhibit astrocyte A1 astrocyte polarization n and reduce its neurotoxicity. Honokiol was able to modulate the SIRT3-STAT3 axis to inhibit STAT3 nuclear translocation and A1 astrocyte polarization, reducing its neurotoxicity (Hu et al., 2023). Previously, Salidroside has been demonstrated to play a neuroprotective role in a variety of central nervous system (CNS) diseases (Zhang et al., 2023; Zheng et al., 2024), and recent studies have shown that Salidroside not only decreases the expression of C3 proteins, but also significantly upregulates axon regeneration factors, such as growth-associated protein 43 (GAP43) and NF200, and the amount of Nestin and Sox2 double-positive stained NSCs, following SCI. Additionally, it also considerably relieves the proliferation inhibition of NSCs induced by A1 astrocytes, facilitating the migration of NSCs to the injured area (Qian et al., 2024). This broadens the prospect of clinical application of Salidroside. Herbal medicines can act on multiple signaling pathways in an integrated manner to achieve a more comprehensive therapeutic effect. However, the capacity to easily cross the blood–brain barrier is also a concern, and further studies are needed to improve the delivery of protective botanicals.

### 4.7 Other therapies

In the MCAO model, cottonseed oil/CSO treatment significantly decreased the number of C3d/GFAP double-positive cells and upregulated C3d protein expression, increased the number of S100A10/GFAP double-positive cells and downregulated S100A10 protein expression, and inhibited protein expression of TLR4 and NF-κB, which in turn inhibited the release of IL-1β, IL-6 and TNF-*α*, and ultimately ameliorated blood–brain barrier disruption and neuronal damage. It is suggested that cottonseed oil exerts neuroprotective effects by reducing neurotoxic astrocyte activation (Liu M. et al., 2020). And recombinant IL-10 (rIL-10) counteracted excessive glutamate release induced by methamphetamine (Meth) in astrocyte cultures, suggesting that rIL-10 may inhibit activation and metabolic levels of A1 astrocytes (Silva et al., 2024).

A large number of treatments have shown great therapeutic promise, and there are reasons to believe that targeted astrocyte polarization will benefit a wide range of SCI patients in the future.

## 5 Summary and prospect

Targeting astrocyte polarization is extraordinarily crucial to functional recovery following SCI. Harnessing their polarization will provide new hope for recovery from SCI. Although significant progress has been made in the regulation of astrocyte polarization, in the future we still need to investigate astrocyte polarization in depth by various means (e.g., genome editing, single-cell RNA sequencing, etc.) to better comprehend the dynamic process of phenotypic transformation, and to identify more new genes involved in astrocyte polarization using high-throughput screening technology to provide a solid theoretical foundation for the development of new therapeutic strategies. Future research should also strengthen preclinical studies to promote adoption for translation into clinical applications. Ultimately, multimodal therapeutic protocols may be employed to regulate astrocyte polarization and promote functional recovery after SCI. With further research and advancements in medical technology, it is expected that multiple interventional strategies targeting astrocyte polarization will be more extensively applied in clinical practice, ultimately improving the motor-sensory function and quality of life of patients with SCI.

It is worth noting that our focus in this article is to review the relevant literature using the A1/A2 phenotype. But in addition to this, there are many authoritative studies that support other methods of typing that are also excellent. However, recent and authoritative studies have shown that when describing astrocyte phenotypes, it is best to avoid vague and binary terms such as ‘neuroprotective’ or ‘neurotoxic’ as they are too simplistic to be meaningful and that there is heterogeneity in the status of astrocytes in different diseases. Astrocyte status is heterogeneous in different diseases. Therefore, multiple criteria should be considered in the future classification of reactive astrocytes, including transcriptomic, proteomic, morphological, and specific cellular functions, as well as the impact on pathological hallmarks (Escartin et al., 2021).
